# Academic Freedom as a Defensive Right

**DOI:** 10.1007/s40803-022-00188-4

**Published:** 2023-01-09

**Authors:** Monika Stachowiak-Kudła, Sina Westa, Catarina Santos Botelho, Ildikó Bartha

**Affiliations:** 1 Collegium of Management and Finance, Warsaw School of Economics, Street Madalińskiego 6/8, 02-513 Warsaw, Poland; 2 University of Warsaw, Krakowskie Przedmieście 32, 00-927 Warsaw, Poland; 3 Erfurt School of Education, University of Erfurt, Nordhäuser Str. 63, 99089 Erfurt, Germany; 4 Porto Faculty of Law, Universidade Católica Portuguesa, Rua Diogo Botelho, 1327, 4169-005 Porto, Portugal; 5 MTA-DE Public Service Research Group and Faculty of Law and Political Studies, University of Debrecen, Street Kassai 26, 4028 Debrecen, Hungary

**Keywords:** Academic freedom, Negative and positive freedom, Defensive rights, Freedom of scientific research, Freedom to teach, Fundamental rights

## Abstract

Ensuring the proper implementation of academic freedom can be difficult both for policymakers and university authorities. Hence, great emphasis should be given to the defensive function of academic freedom. In this paper, we analyse the legal regulations and the jurisprudence of the constitutional courts of Germany, Hungary, Poland, Portugal, and Spain. We identify who is the holder of academic freedom, how the defensive function of academic freedom works and what academic activities are being protected. The study shows that individual countries emphasise slightly different aspects of the defensive function of academic freedom but remain unanimous on the essence of this function. As academic freedom is not defined unequivocally in most constitutions and legal frameworks, constitutional courts play a significant role in shaping its defensive function.

**Supplementary Information:**

The online version contains supplementary material available at 10.1007/s40803-022-00188-4.

## Introduction

Higher education communities perform several important and utilitarian roles in society: develop valuable skills and services, train a highly skilled workforce, foster economic development, and conduct research.^1^ Higher education communities are a model example of how democratic value systems work, particularly the democratic ‘knowledge-over-force’ principle that rejects violence and force as determinants of outcomes. They are models and teachers of democratic values.^2^ To fulfil their role properly, these higher education communities must be based on fundamental values such as equality of access to higher education, the autonomy of higher education institutions, responsibility, and quality, as well as academic freedom.

Academic freedom has many definitions.^3^ Its meaning is based on history and culture and it can change across time and regions.^4^ In this article, we will deal with academic freedom which is understood as ‘the right, without constriction by prescribed doctrine, to freedom of teaching and discussion, freedom in carrying out research and disseminating and publishing the results thereof, freedom to express freely their opinion about the institution or system in which they work, freedom from institutional censorship and freedom to participate in professional or representative academic bodies’.^5^ Academic freedom is guaranteed by Article 13 of the *Charter of Fundamental Rights of the European Union* (EU Charter)^6^ and by the constitutions of many countries in which it appears in the form of freedom of scientific research and/or freedom of teaching.^7^ It protects scientific and teaching activities against unjustified interference from public authorities and/or universities and also from other academics. This defensive function of academic freedom is essential for the creation of knowledge.^8^

Ensuring the proper implementation of academic freedom can be difficult, with challenges arising from several factors. When higher education is dependent on one key source of funding (usually the state or the taxpayer),^9^ financial sponsors of higher education institutions may expect ‘appropriate’ academic knowledge or may be able to block results that are inconvenient or too revealing.^10^ Obstructing results does not have to be the domain of financial sponsors alone but can be practiced by influential corporations.^11^ The introduction of managerial approaches to university governance can be a factor of pressure to conduct ‘safe’ research, where controversial research will be considered undesirable.^12^ This model strengthens the traditional intra-community focus around traditional disciplines and thereby negatively affects interdisciplinary research.^13^ Due to the strong emphasis on the scientist being ‘excellent at an international level’, the managerial approaches to university governance can marginalise research of local importance.^14^ However, the state can restrict academic freedom not only as a funder but also by arbitrarily shaping the legal conditions for university accreditation in order to marginalise certain political views.^15^ Sometimes we witness attempts to put pressure on higher education institutions in order to silence academics or students speaking or acting against political consensus.^16^ Additionally, we may observe a conflict between freedom of academic expression and any prevailing orthodoxy of political correctness.^17^ The political sphere also exerts a clear but complex influence on the degree to which academic activities are free.^18^ Under such circumstances, in which the proper safeguard of academic freedom is at risk, the need to clarify the defensive nature of academic freedom is vital.

This paper aims to clarify three important questions. It shows (1) who is the holder of academic freedom, (2) how the defensive function of academic freedom works, and (3) what activities carried out under this freedom are subject to protection. We decided to answer these questions by analysing the legal regulations and case law of the constitutional courts of Germany, Hungary, Poland, Portugal, and Spain, and these countries were chosen for two reasons.

First, we assume that constitutional courts in countries where the tradition of academic freedom has been interrupted by periods of undemocratic regimes will more often pay attention to the defensive function of academic freedom than those in countries without such experience. Hungary, Poland and partly Germany (in the territory of the former German Democratic Republic) are all countries that transitioned to democracy after an extended period of ‘real socialism’,^19^ and had to transform academic institutional systems in which academic freedom was not enforced. For all of Germany, the experience of World War II was significant, and triggered the need for a strong normative Basic Law that would prevent a repetition of National Socialism. After periods of military right-wing dictatorships and authoritarian regimes that lasted until the 70’s, Spain^20^ and Portugal^21^ also reformed their higher education systems towards increasing university autonomy and guaranteeing academic freedom. The constitutional guarantees of academic freedom were of particular importance in these democratic transitions, however, we also have an example of a country among those selected (Hungary) where these democratic achievements have once again come under threat (since 2010). Such a turn is also reflected in the adoption of a new constitution in Hungary,^22^ therefore we make a distinction between ‘pre-2012’ and ‘post-2012’ legislation and constitutional practice in this study.^23^

Our original database includes all 105 judicial decisions (see appendix) from eight European countries fulfilling two criteria: they have constitutional courts, and they provide constitutional regulations protecting academic freedom or the right of universities to autonomy (Belgium, Czechia, France, Germany, Hungary, Poland, Portugal and Spain).^24^ Due to the fact that the constitutional courts of some of these countries have not expressed their views on the defensive function of academic freedom or have spoken in a very narrow range, the number of countries analysed was limited to five. Among the countries selected for further analysis, we identified 77 decisions of constitutional courts referring to freedom of scientific research or freedom of teaching. However, only 23 cases related to the direct violation of these freedoms, most of them from Germany (9 cases). In other cases, academic freedom is cited in the background of a dispute about a violation of another right, most often the right of university autonomy.

Second, the constitutional jurisprudence of the five selected countries represents the three distinct waves of constitutional adjudication in post-war Europe that make them interesting for comparative analysis.^25^ These countries are all members of the European Union and the European Higher Education Area and therefore are also obliged by the norms, values and guiding principles arising from such memberships in the area of academic freedom.^26^

In this paper, we focus on the perception of academic freedom by judges of constitutional courts. The constitutional courts play an active role in the legal protection of fundamental rights and, due to their above-mentioned function, more often face the issue of academic freedom than other courts. Analysing constitutional courts’ understanding of academic freedom and its defensive function seems to be important nowadays when many universities around the world, including in Europe, face serious threats to their institutional autonomy.^27^ We discuss the legal literature on academic freedom and the literature on the developing theory of constitutional rights of Robert Alexy and the theory of negative and positive freedom of Isaiah Berlin.

The paper is structured as follows. After introducing the key problem, the second section delineates academic freedom as an example of a fundamental freedom. The third section explains who is the holder of academic freedom. The fourth part offers insight into how the constitutional courts of five selected countries understand the defensive function of academic freedom. The final section briefly concludes the findings of the paper.

## Academic Freedom as a Fundamental Freedom

Academic freedom belongs to the group of fundamental freedoms, that is, those that have been recognised as requiring a high degree of protection from governmental encroachment.^28^ Freedom is ‘the absence of obstacles to possible choices and activities’.^29^ The fundamental rights and freedoms are contained in the constitutions of particular countries. If fundamental rights or freedoms are not explicitly stated in the constitution, constitutional courts may infer them from other rights. Thus, academic freedom can be derived from freedom of opinion and expression and from the right to education.^30^ Similarly, we can observe this in Article 10 of the *European Convention of Human Rights*, in Article 19 of the *International Covenant on Civil and Political Rights* and in Article 13 of the *International Covenant on Economic, Social and Cultural Rights*.

### Positive Definitions of Academic Freedom

Only some of the surveyed countries adopted a legal definition of the rights constituting academic freedom. The legal definition of the freedom of teaching can be found in German laws, whereas the legal definition of the freedom of scientific research can be found in German and Portuguese laws (see Table 1, columns A and B).

**Table 1:** Academic freedom in legal acts and jurisdiction

	A	B	C	D	E	F
Germany	Yes	Yes	No	Yes	Yes	Yes
Hungary	No	No	Yes	Yes	No	Yes
Poland	No	No	No	Yes	No	Yes
Portugal	No	Yes	Yes	Yes	No	Yes
Spain	No	No	No	Yes	Yes	Yes

**A**—countries which have a legal definition of freedom of teaching;

**B**—countries which have a legal definition of freedom of scientific research;

**C**—countries that refer to the defensive function of academic freedom in the constitution;

**D**—the constitutional court has ruled that academic freedom protects the individual from unjustified interference from public authority;

**E**—the constitutional court has proclaimed that academic freedom protects the individual from unjustified interference not only by the part of the state, but also against any interference, including interference by university / faculty authorities and students;

**F**—the constitutional court has decided that academic freedom has both a negative and a positive dimension

Source: Own survey of acts regulating higher education and jurisdiction in the constitutional courts of the selected EU countries. Legal status revised at the beginning of 2021

Dieter Grimm indicates that the fundamental right provision most often ‘declares a certain conduct (e.g., the expression of a certain opinion) or a certain state (e.g., physical integrity) or certain social institutions (e.g., media, science, arts) to be “free”. At the same time, it empowers the legislature to limit this freedom'.^31^ This is clearly visible in the case of Basic Law for the Federal Republic of Germany: ‘Arts and sciences, research and teaching shall be free’ (Article 5(3)),^32^ but not for the other countries surveyed. In Germany, the *Framework law on higher education* states that the freedom of research ‘shall in particular include posing questions, applying methodological principles, as well as evaluating and sharing research results’. The freedom of teaching ‘shall in particular include the teaching of courses, the creation of the courses' content, the methods of instruction, as well as the right to express artistic views and academic opinions’. The freedom of study shall include the right to freely choose courses, to set one’s own study emphasis within a degree program as well as to work out and to express one’s own scientific and artistic opinion. The university is allowed to make decisions on these matters.^33^

The Federal Constitutional Court has supplemented the above definitions by pointing out that protection extends to research-based teaching as a process of transferring scientific knowledge.^34^ Protection is granted in particular to the self-determination of the content, procedure and methodological approach of the course^35^ and to the expression of scientific opinions,^36^ and the right to participate actively in scientific discussion during the course of studies.^37^ Furthermore, it clarifies that academics need to be involved in the organisation and governance of a university in such a way that inadequate scientific decisions are prevented and that freedom in research and teaching is guaranteed.

In this line, policy makers need to provide a satisfactory possibility for participation in higher education governance to ensure the execution of academic freedom.^38^ The essence of academic freedom for teachers in higher education is the right to represent their own subject in research and teaching. In the case that there are state interferences in a certain academic aspect, Article 5(3) of the Basic Law shall be followed. This means it is allowed to establish faculties of theology at state universities.

In Hungary, the wording of Sect. 70/G of the Constitution in force between 1989 and 2011 stated that: ‘the Republic of Hungary shall respect the freedom of scientific life’. The current Constitution of 2011 states in Article X(1) that ‘Hungary shall protect the freedom of scientific research and artistic expression, as well as the freedom of learning and—within the framework defined by law—teaching so as to attain the highest level of knowledge possible’. In Hungary, while the former *Act on Higher Education* (in force until 2011)^39^ expressly mentions the achievement of ‘the freedom to learn, teach, and do scientific research … within the operation of the education system and individual institutions’ among the fundamental objectives of the act (but without specifying the content of such freedom), the current *Act on National Higher Education* does not include the academic freedom guarantee^40^ at all.^41^ However, the Hungarian Academy of Sciences also plays a key role in scientific life in this country due to its extensive research network. Therefore, the wording of the *Act on the Academy* may be relevant, the preamble of which refers to freedom of scientific research without specifying exactly what it is.^42^

The essence of academic freedom has been defined by the Constitutional Court of Hungary. In its pre-2011 decision,^43^ this court ruled that the freedom of scientific research includes the right to conduct scientific research and to disseminate scientific truth and knowledge.^44^ It also confirmed that:

‘there is a coherent link between freedom of information and freedom of academic knowledge, academic research and teaching. ... by guaranteeing the freedom to obtain information, the constitution indirectly guarantees and protects the freedom of academic knowledge which is part of this freedom.’^45^

The Constitution of the Republic of Poland states that: ‘the freedom of artistic creation and scientific research as well as dissemination of the fruits thereof, the freedom to teach and to enjoy the products of culture, shall be ensured to everyone’ (Article 73). Neither the Constitution of the Republic of Poland nor the *Act—Law on Higher Education and Science* explain the essence of these freedoms.^46^ The scope of this freedom has been defined by the Constitutional Court. This Court interprets the essence of freedom of scientific research as the freedom to choose subjects for scientific research, freedom to choose methodology, and freedom to publish the results. The Constitutional Court in Poland has also ruled that ‘the freedom to access all information that may be needed for the research’ is an element of the freedom of scientific research.^47^ ‘Freedom to teach, in turn, includes the freedom of systematic transfer of knowledge to other people’.^48^ Due to the difficulties in ensuring academic freedom by Polish higher education institutions, the *Act—Law on Higher Education and Science* was amended in 2021. This indicated that the Rector's tasks include, ‘ensuring that the university respects the freedom of speech, teaching, research and the publication of their results, as well as academic debate organised by members of the university community, in compliance with the principles of world-view pluralism and university order regulations’ (Article 23(2a)).

The Constitution of the Portuguese Republic proclaims that: ‘there shall be freedom of intellectual, artistic and scientific creation’ (Article 42) and that ‘the freedom to learn and to teach is guaranteed’ (Article 43). The Portuguese Constitution is the only constitution analysed in this article which includes a direct definition of academic freedom. It prescribes that the freedom of scientific research includes the right to create, produce and distribute scientific works, and guarantees statutory copyright protection.^49^

The Spanish Constitution states that: ‘the following rights are recognised and protected: … the right to academic freedom’ (Articles 20(1c)). The *Act—Law on university coexistence* does not explain the essence of this freedom.^50^ The Constitutional Court of Spain has declared that freedom of teaching is related to ‘the right to freely disseminate the thoughts, ideas and opinions of teachers while performing their functions’.^51^ This Court states also that: ‘academic freedom is essential to the creation, development, transfer and critique of science, technology and culture’.^52^

The theoretical contribution that can be made from this analysis of the different cases is as follows: fundamental rights rarely have a legal definition. If such a definition exists, it is contained in an act rather than in the constitution. The lack of a legal definition means that in the event of a dispute, administrative courts and the constitutional court determine what the essence of these freedoms is and what it is not. The judges apply the governing law to the facts of a case.^53^ In practice, this means that the judge does not have to refer to all aspects of academic freedom, but only to those that are relevant to the resolution of the dispute. The courts supplement the definition of academic freedom by presenting the relationship of this freedom with other rights set out in the constitution.

### The 'Negative Side' of the Definition of Academic Freedom

The definitions presented above are positive definitions of academic freedom. The full definition of a fundamental freedom should also include the ‘negative side’, which would indicate what cannot be done with, and through, such rights.^54^ Dieter Grimm believes that the fundamental rights and freedoms are those whose extent ‘can only be ascertained by knowing the statutes which limit this freedom’.^55^ Robert Alexy indicates that ‘Many defensive-rights provisions contain an authorisation of the legislature to limit its enjoyment. Such an authorisation gives the legislature the constitutional power to decide for itself, and on the basis of, which goals and policies it wishes to limit the enjoyment of the right. This power of limitation is, naturally, limited by the principle of proportionality, but within these limits it is an instance of discretion’.^56^

Some constitutional texts contain a provision relating to the possibility of limiting rights and freedoms, including academic freedom. This is the so-called ‘limitation clause’,^57^ an example of which can be found in Article 31(3) of the Constitution of Poland, which states that ‘any limitation upon the exercise of constitutional freedoms and rights may be imposed only by statute, and only when necessary in a democratic state for the protection of its security or public order, or to protect the natural environment, health or public morals, or the freedoms and rights of other persons. Such limitations shall not violate the essence of freedoms and rights.’ In the case of academic freedom, the Federal Constitutional Court of Germany,^58^ and the Constitutional Courts of Poland^59^ and Spain^60^ clearly prefer the limitation clause based on the protection of other individual rights.

In addition to the protection of individual rights, the Federal Constitutional Court of Germany has indicated that interference with the freedom of scientific research is justified if its purpose is to ensure the quality of teaching.^61^ The enjoyment of academic freedom in Germany is also restricted by the Basic Law where ‘the freedom of teaching shall not release any person from allegiance to the constitution.’^62^ A particular restriction of academic freedom applies to teachers of theology in higher education as they shall be restricted by the autonomy of religious communities and the right of the faculty to shape their identity as a theological faculty.^63^

The constitutional Court of Poland gives examples of rights that should not be violated by those exercising the freedom of scientific research: the right to dignity, privacy and freedom of conscience and religion.^64^

The Constitutional Court of Spain have clearly pointed to the limitations of academic freedom resulting from the necessity of the protection of other individual rights. This court has also pointed out that academic freedom is not the right to fully and independently regulate the teaching function irrespective of the organizational criteria established by the management of a university centre.^65^

## Holders of Academic Freedom

Academic freedom is a freedom closely related to members of the institutions of higher education and science.^66^ Early academic freedom researchers have already pointed out that it is a response to the social contribution of expert professors and researchers.^67^ This view still has many supporters today.^68^ Hence, academic freedom should be defined as a fundamental freedom rather than as a human right. The latter are natural rights, thereby, pre-legal rights that apply to people simply by virtue of being human.^69^ Academic freedom is the right of everyone only in Poland, thus it can be defined as a human right, whereas in other countries it is the right of every single researcher, academic teacher and student, and hence it is a fundamental right in these cases. In the following, we will show exactly who is entitled to academic freedom in the different cases as may be prescribed by law.

In Germany, as already mentioned, Article 5(3) of the Basic Law states ‘arts and sciences, research and teaching shall be free.’^70^*The University Framework Act* states precisely for whom this freedom applies. In paragraph 4(1), it is defined that the country and higher education institution are obliged to safeguard academic freedom and that members of the higher education institution are able to exercise the freedom given by the Basic Law of Germany Article 5(3), clause 1. In paragraph 36, it defines a member of the university as either a person who works mainly for a higher education institution (not only temporally or as a guest) or a student enrolled in the higher education institution. Paragraph 58 guarantees autonomy for higher education institutions.^71^

The Constitution of Poland clearly indicates (Article 73) that everyone is entitled to the freedom of scientific research and the freedom of teaching. The Constitutional Court of Poland states that these freedoms can also be ‘conducted outside of the academic community without being in relation to a university.’^72^ Importantly, in the said judgment, the Constitutional Court of Poland also states that: although the legislator has broadly defined the group of entities using this freedom, stating that it applies to ‘everyone’, there is no doubt that the freedom of scientific research, freedom to publish research results and freedom of teaching are particularly important for academics.^73^ In Poland, academic freedom is therefore a freedom for every person.

In Hungary, Portugal, and Spain, in the face of the silence of the laws, it is the constitutional courts that have indicated who is protected by academic freedom. In its pre-2011 decision, the Constitutional Court of Hungary stated that ‘the right to freedom of scientific life is in principle enjoyed by all, but the actual holders of this right are only those who practice science.’^74^ Potentially, everyone is entitled to this right, but participation in scientific life is a precondition for entitlement. These rights also belong to teachers, researchers, and students.^75^

In Portugal, the Constitutional Court points to the professors who personally exercise their freedom of research and teaching.^76^

The Constitutional Court of Spain identifies both *la libertad de cátedra*^77^ and *la libertad de enseñanza*^78^ as the rights of academic teachers, i.e. those who personally perform the action of teaching at a university.

Some jurisdictions also make another distinction, namely whether an academic works in a public or private institution. In the United States, for example, academic freedom is only available to academics in state universities, whereas private universities are subject to such obligations only as a matter of voluntary self-regulation.^79^ However, such a distinction is not made in the legislation of any of the countries examined in this paper.

In the jurisprudence of constitutional courts, academic freedom is often invoked with the right of universities to autonomy. The holder of ‘the university's right to autonomy are the university's constitutive and executive bodies.’^80^ Therefore, this right is implemented by the university authorities, not by a single researcher or academic teacher.

However, in Germany, where the constitution does not guarantee a university's autonomy, the Constitutional Court derives this right from freedom of teaching.^81^ The fact that the holder of academic freedom is a different entity than the university's right to autonomy can be seen in the case of a collision of rights. According to Robert Alexy's theory,^82^ fundamental rights and freedoms, which also include academic freedom, are also principles and may collide with other rights. The collision between two or more rights are settled by constitutional courts and international tribunals by applying the principle of proportionality.^83^ The constitutional courts in Germany^84^ and Spain^85^ have resolved the collisions of the university's right to autonomy with academic freedom. In these judgments, the courts understood university autonomy as a separate right, not another aspect of academic freedom.^86^

## The Defensive Function of Academic Freedom

### Negative and Positive Dimensions of Academic Freedom

**Fig. 1:**
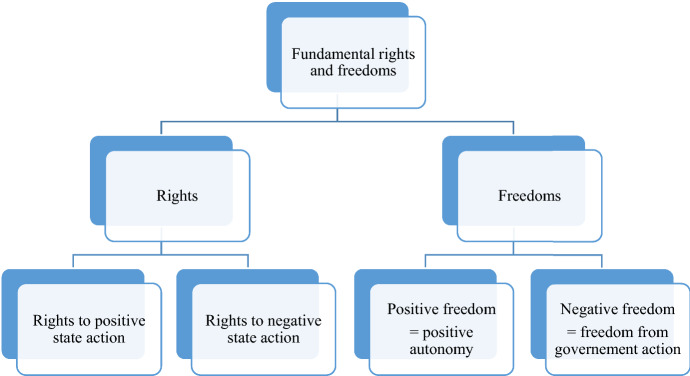
Fundamental rights and freedoms

Isaiah Berlin in his famous essay, *Two concepts of liberty,* popularised the division between negative freedom and positive freedom (Fig. 1). Negative freedom is defined as ‘freedom from’ external interference; it is about the absence of constraints,^87^ or, as it is sometimes put, ‘the right to be left alone’. As a negative freedom, academic freedom means freedom from government action. To enforce a negative freedom, an academic merely insists that the government not act to impinge his/her freedom. Positive freedom is known as the 'freedom to’ do something, including the ability and opportunity to do so.^88^ Kai Möller believes that ‘positive freedom is synonymous with autonomy, in particular personal autonomy’. Joseph Raz claims that ‘the ruling idea behind the ideal of personal autonomy is that people should make their own lives.’^89^ Charles Taylor says that negative freedom denotes mere power or opportunity and that positive freedom refers to actual self-realisation or achievement.^90^ Gerald MacCallum notes that the differences between negative and positive freedom concern the range of agents, preventing conditions and actions involved in the statement ‘x is (is not) free from y to do (not do, become, not become) z.’^91^

**Fig. 2:**
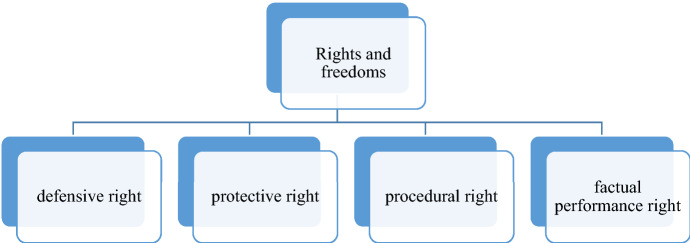
Rights and freedoms in the concept of division of entitlements

Fundamental rights ‘are designed in the first instance to secure a sphere of liberty for the individual from interferences by public power; they are defensive rights of the citizen against the state.’^92^ In the concept of division of entitlements of Robert Alexy (Fig. 2) defensive rights are a prohibition against destruction. Robert Alexy indicates that ‘when there is a prohibition on destroying or adversely affecting something, then every act that represents or brings about destruction or an adverse effect is prohibited.’^93^ Dieter Grimm defines ‘infringements’ on fundamental rights ‘as encompassing all state actions that preclude, or substantially impede, the enjoyment of a fundamental right.’^94^ In this concept, academic freedom is:

- a right against the state that it should not obstruct acts within the field of academic life (defensive right),
- a right that the state protect the right-holder from academic freedom damaging acts of third parties (protective right),
- a right that the state allows the right-holder to be involved in decisions affecting academics (procedural rights) and
- a right that the state undertakes certain actions to exercise this right (factual performance right).^95^

In this line of argumentation, the German Federal Constitutional Court proclaims that the essence of the freedom of research is the lack of state interference in conducting the research and the process of publishing the results.^96^ Moreover, the German Federal Constitutional Court ruled that constitutional rights do not only apply as defensive rights against the state, but also have a horizontal effect as they represent value orders that apply to all aspects of the law.^97^ Furthermore, the Court points out the need to protect academic freedom from interference by the university or faculty authorities^98^ or students. Hence, it indicates that, ‘individual lectures are to be protected from active boycotts by audiences or from third parties who attend simply to disrupt.’^99^

The defensive function of academic freedom is also emphasised in the Hungarian Constitution (both in the previous version and the current Fundamental Law): ‘the state shall have no right to decide on questions of scientific truth; only scientists shall have the right to evaluate scientific research.’^100^ At the same time, this defensive function (or certain parts of it) may also be weakened by the constitutional document itself. In Hungary, the Fourth Amendment to Fundamental Law in 2013^101^ served as a basis for several modifications to the Act on National Higher Education that reduced financial and institutional aspects of academic freedom considerably,^102^ and in doing so, significantly helped to enforce government preferences in subjects and directions of academic research.^103^ According to the pre-2011 practice of the Hungarian Constitutional Court, the freedom of science requires from the state the guarantee of the autonomy of science.

The freedom of science is an aspect of the right of communication, from which the Court has derived the special protection of the autonomy of science and the right of those who are engaged in science, to make decisions concerning science.^104^ It was in light of these principles that, in 2005, the Constitutional Court of Hungary declared the establishment of the so-called ‘governing councils’ unconstitutional, independent of higher education institutions but under the influence of ministers, which contributed to the definition of the scientific research agenda. It also found it unconstitutional for the government to determine the disciplines in which PhD courses could be offered, as well as for the Minister of Education to restructure or dissolve a higher education institution^105^ in certain cases.^106^

The Constitutional Court of Poland, settling the dispute over the essence of the right to social security, states that academic freedom ‘protect[s] the individual and other law entities from ungrounded state interference in the subject and methods of scientific research and in the content and methods of teaching’. Moreover, it has highlighted that this right does not constitute grounds for academics to make any claims regarding their material status.^107^

The defensive function of academic freedom is emphasised in the Portuguese Constitution: ‘the s*tate may not program education and culture in accordance with any philosophical*, *aesthetic*, *political*, *ideological or religious directives*.’^108^ The Portuguese Constitutional Court, citing the Portuguese scholar José Casalta Nabais, emphasises that.

‘universities […] will only be autonomous to the extent that they are acknowledged to possess a domain of their own interests (affairs)—a domain in relation to which the state administration restricts itself to exercising a merely coordinating form of oversight; i.e. to the extent to which they constitute something more than mere instruments (albeit equipped with a public-law persona—albeit public institutes) of the state’s indirect administration.’^109^

Similarly, the Spanish Constitutional Court, deciding on a matter concerning the scope of the statutory autonomy of universities, points to the protection of academic freedom against all public powers.^110^

Similarly, the Spanish Constitutional Court points out the need to protect academic freedom from interference by faculty authorities. The Court states that an academic teacher does not have to represent the views preferred by the authorities of the institution in which he/she/they works.^111^

The defensive function of academic freedom is all the more important because some countries have already decided to emphasise it in the text of their Constitution (see Table 1, column C). In the case of academic freedom, its protection against state interference is strongly emphasised. This is clearly visible in the jurisprudence of constitutional courts (see Table 1, column D). Most often, violation of academic freedom consists of preventing academics from conducting scientific research, publishing their findings, delivering academic lectures and travelling to international scholarly meetings.^112^ Violations of academic freedom, which interfere with this right of academic administration, most often manifest themselves in restricting the freedom of academic staff to assign students’ grades at their own discretion.^113^ Academic freedom protects scientific and teaching activities against the interference of not only the public authorities and the general public but also other authorities, including university and faculty authorities.^114^ Some of the courts investigated point to the need to protect academic freedom from interference by the university or faculty authorities or students (see Table 1, column E).

### 'Positive' State Obligations Towards the Protection of Academic Freedom

Academic freedom is also a value or, as Robert Alexy^115^ describes, a principle that gives impetus and provides guidelines to all areas of the law to which it is relevant. The constitutional courts of the countries examined in this paper indicate that the protection of academic freedom requires positive action by the state (see Table 1, column F). The right to positive state action requires not only that the state forbear from interfering in the spheres they protect, but also to take measures to protect the interests protected by rights against violations, especially by third parties.^116^

The German Federal Constitutional Court points to the obligation of the state to protect and support academic freedom, among other things by ensuring appropriate measures, including organisational measures,^117^ as well as the legislator's obligation to ensure a sufficient level of participation of freedom of scientific research in university bodies.^118^

The positive dimension of academic freedom has recently been challenged in a case concerning Hungary’s higher education system, initiated before the Court of Justice of the European Union (CJEU). The CJEU confirmed the member state's obligation to protect academic freedom by creating a legal and organisational framework for the functioning of higher education institutions. It established that ‘academic freedom also incorporates an institutional and organisational dimension, a link to an organisational structure being an essential prerequisite for teaching and research activities.’^119^

The CJEU made this finding in relation to the amendment to the National Higher Education Act of Hungary in 2017, which had made the operation of foreign accredited higher education institutions in Hungary conditional to the conclusion of an international treaty between Hungary and their state of origin, and proof that higher education was also being offered in their state of origin.^120^ The Central European University (CEU) seemed to be expressly explicitly targeted by this amendment as it was the only one (of the six institutions affected by the amendment) that was unable to fulfil the new requirements. The CJEU held that the amendment of 2017:

‘is capable of endangering the academic activity of the foreign higher education institutions concerned within the territory of Hungary and, therefore, of depriving the universities concerned of the autonomous organisational structure that is necessary for conducting their academic research and for carrying out their educational activities.’^121^

Consequently, it constituted a limitation of the academic freedom protected in Article 13 of the EU Charter.^122^

However, the uniqueness of academic freedom lies within the fact that the obligation to create conditions for the implementation of this right rests primarily not with the state, but with the universities. In order for universities to be able to fulfil this obligation, they are granted the right to autonomy.^123^ The Constitutional Court of Hungary declared in 2005, that ‘freedom of science is realised through the rights of self-government granted by the state to higher education institutions established under its duty to protect institutions.’^124^

In 2021, the Hungarian Constitutional Court overruled its previous decision from 2005 and reinterpreted the content of university autonomy. In the proceedings launched by the court’s initiative, the initiating judge took the view that a regulation whereby the funding body completely deprives the university senate of its organisational and economic autonomy^125^ is unconstitutional, would lead to the erosion of university autonomy and would infringe academic freedom. The Constitutional Court, however, rejected the judicial initiative on the reasoning that the autonomy of higher education institutions concerns the content of academic research and teaching and the funding body has no decision-making power regarding these matters. According to the Court, organisational and economic autonomy is not necessary to guarantee academic freedom, only the freedom of expression of academics, lecturers and students.^126^ The fact that the senate does not have the right to decide on the budget and rules of the organisation's operations does not violate university autonomy if it has the opportunity to express its opinion on these matters.^127^ At the same time, the Court declared, as a constitutional requirement arising from the Fundamental Law of Hungary, that:

‘the funding body must allow sufficient time for the senate of the higher education institution to exercise its right to express its views, as the guarantor of the higher education institution's autonomy in teaching and research, and must provide the opportunity for preparing substantive proposals, which the funding body must take into account in a transparent manner in its decision-making’.^128^

However, a subsequent decision of the Hungarian Constitutional Court reduced this constitutional requirement into an empty shell by declaring a government decree, adopted as an emergency measure during the Covid-19 pandemic, as not violating academic freedom.^129^ The decree grants *exclusive power to the funding body* (without giving an opportunity to the senate to express its opinion) to establish, in the event of a public health or safety emergency, that the conditions for the fulfilment of the students’ academic obligations, are not met.^130^

The Polish Constitutional Court, ruling on the constitutionality of the introduction of payment for part-time studies, states that ‘the autonomy of higher education aims to create conditions for these institutions to optimally implement their tasks in the field of research and teaching.’^131^

The Portuguese Constitutional Court provides the reminder that:

‘the autonomy of universities has established itself over time, essentially and above all as the freedom to think, conduct research and teach; but this is a freedom which is institutionalised, within the social community, or is exercised, in an objective manner, by a specific scientific *corpus* by acknowledging in Article 76(2) that universities possess the autonomy to decide their own articles of association and scientific and pedagogical, administrative and financial autonomy. The[Portuguese] Constitution, in fact, enshrines the axiological/historical core of that which truly identifies them: *institutions* which practice the freedom to think and conduct research, which base their activities on that freedom, and which transmit the knowledge that is obtained in this way to both university students and the social community.’^132^

Furthermore, the Portuguese Constitutional Court adds that ‘there is no doubt that the rule laid down by Article 76(2) of the Constitution sees university autonomy as a fundamental guarantee whose subjective extent goes beyond the mere institutional level. It also projects itself to some extent into the sphere of university agents—particularly in regard to freedom of research, teaching, thought, and pedagogy—always in compliance with the Constitution and embracing that which is customarily called the “freedom of professorship”.^133^

The Spanish Constitutional Court, by resolving a dispute over the right of an academic teacher to teach the subject in which he or she is a specialist, states that:

‘academic freedom, as individual freedom of the teacher, is a projection of ideological and religious freedom and the right to freely disseminate the thoughts, ideas and options of teachers in the exercise of its function. It consists, therefore, in the possibility of expressing the ideas or convictions that each teacher assumes as their own in relation to the subject matter of their teaching. In this regard, as the right of each teacher, academic freedom has a predominantly negative content in that (STC 5/1981) it “enables the teacher to resist any mandate to give the teaching a determined ideological orientation”, and is a notion incompatible with the existence of any official doctrine, since it supposes the non-subjection of the teaching activity to any system of values, except those enshrined by the constitutional legal order itself. The academic freedom coexists with a positive dimension: ‘university autonomy is the institutional dimension of academic freedom, which guarantees and completes its individual dimension, constituted by academic freedom. In this way, academic freedom, as an individual right of each teacher, presupposes and requires the organisation of teaching and research attributed to the University itself by virtue of its autonomy.’^134^

The protection of academic freedom, as the purpose of university autonomy, is also indicated by the Spanish Constitutional Court: ‘autonomy is the institutional dimension of academic freedom that guarantees and completes its individual dimension. Both serve to delimit that, “space of intellectual freedom” without which “the creation, development, transmission and criticism of the science of technology and culture”, is not possible’.^135^

The theoretical contributions that can be made from this analysis are as follows: academic freedom is a defensive freedom and a negative freedom that also requires governmental action to meet constitutional requirements realistically. David Sklansky has termed such a right as a ‘quasi-affirmative right’.^136^

## Concluding Remarks

Academic freedom is a fundamental freedom and as such is protected in both a positive and negative context. Different countries emphasise slightly different aspects of the defensive function of academic freedom. The differences result from factors related to distinct law traditions, educational culture, state-university relations, as well as the relevant historical and political contexts. The emphasis by constitutional courts on various aspects of academic freedom is also directly related to the essence of the dispute being resolved. There is, however, a certain consensus that the defensive nature of the right means that this right defends its holder from interference with his or her rights by the state and university or faculty authorities. University autonomy in this respect plays a crucial role. In practice, this means that higher education institutions cannot invoke their autonomy to limit the academic freedom of an academic. It is recognised that the essential content of university autonomy, defined as a fundamental right, is made up of the elements necessary to ensure respect for academic freedom. Seen as a whole, the manner of protection of academic freedom must not render the requirement of protection illusory.

Effective judicial protection of academic freedom is essential because the content of academic freedom and the nature of its defensive function, especially as manifested in constitutional documents and higher education laws, can be shaped by the governing parliamentary majority according to its own particular goals and interests. Accordingly, the constitutional guarantees of academic freedom have been particularly important in democratic transitions, and this approach has been reflected in the text of the relevant norms adopted in the selected countries, as well as in the case law of constitutional courts (both in the subject matter of cases and the arguments brought and highlighted in the reasoning). The importance of these guarantees is also illustrated by the cases that clearly show the consequences of a parliamentary majority weakening the legal basis for the constitutional protection of academic freedom (see the developments in Hungary after 2011).

The government’s primary function from a constitutional standpoint should be to ensure that deprivations of constitutional rights are avoided as it pursues its policy agenda.^137^ Berggren and Bjørnskov proved by conducting an empirical analysis of 64 countries across the world during the past half-century, that the role of government and judicial institutions in securing academic freedom is significant.^138^ The key result of their research is that democratization is positive for academic freedom. They have found a positive effect on the academic freedom of legislatures, which are becoming more diverse and moving ideologically to the right.^139^ Our study confirms this relationship in the case of Poland, in which the conservative government strengthened academic freedom by pointing out that the Rector's tasks include ensuring this freedom at the university.

Nevertheless, as mentioned, we also have an example among the selected countries (Hungary) where these democratic achievements have once again come under threat since 2010. In this country, the political turn has not only been followed by legislation (a new constitution and the higher education act as well as their subsequent amendments) adversely affecting the level of academic freedom but also reflected in the post-2012 case law of the Hungarian Constitutional Court.^140^ A similar phenomenon cannot be observed in the constitutional jurisprudence of the other examined countries.

Academic freedom is a freedom that only applies to a certain group of people defined by law, and depending on the national context, includes different higher education members, except in Poland where the right extends to everyone (Article 73 of the Constitution). This assumes that there are certain traits connected to members of higher education institutions that need a special project alongside the general freedoms of all people. Academic freedom is a response to the social contribution of professors and research experts. They should not only be protected from interferences of the state but also from the governing bodies of the employing institution.

What becomes obvious is that academic freedom, like many other fundamental rights and freedoms, is not defined unequivocally in most constitutions and/or legal frameworks. In other words, in the countries that we have analysed in this paper, the concrete definition and demarcation from other rights depends not only on the existing law but is framed by the decisions of constitutional courts. In order to secure the application of academic freedom, it not only requires action from government and higher education institutions as we have shown, but also an awareness of the right among its beneficiaries. Only through people who put forward complaints and requests against the violation of academic freedom, will this right be framed more precisely by constitutional courts. Precise knowledge on who is protected by academic freedom and on how its defensive function is understood is the basis for providing legal certainty for high-quality academic work. Thus, making the legal dimension of academic freedom transparent for members of higher education institutions is a worthwhile endeavour. Only through this knowledge are the beneficiaries of academic freedom capable of identifying situations in which it is infringed, and based on this study, are they are able to take legal action.

## Supplementary Information

Below is the link to the electronic supplementary material.

Supplementary file1 (XLSX 16 KB)

## Data Availability

Court sentences are available at constitutional courts websites: https://alkotmanybirosag.hu/ugykereso (Hungary); https://www.bundesverfassungsgericht.de/SiteGlobals/Forms/Suche/Entscheidungensuche_Formular.html?language_=de (Germany); https://ipo.trybunal.gov.pl/ipo/Szukaj?cid=1 (Poland); http://www.tribunalconstitucional.pt/tc/acordaos/ (Portugal); http://hj.tribunalconstitucional.es/es/Busqueda/Index (Spain). The dataset is attached at the end of the paper as an excel file.

## Footnotes

1 Gumport 2000, p. 70. See also: Szadkowski and Krzeski 2021

2 Quinn and Levine 2014, pp. 898–920; Post 2012, pp. 27–43.

3 Altbach 2001, pp. 205–219.

4 Marginson 2014, pp. 24–41; for the historical aspects of higher education in Europe see for example: Rüegg 1993, 1996, 2004, 2010; in Germany see for example: Boockmann 1999, in Hungary see: Ladányi 1999; Bazsa 2014, pp. 47–65; Kováts et al. 2014, in Poland see: Stachowiak-Kudła 2022, pp. 99–111, in Portugal see: Amaral and Carvalho 2003, pp. 35–46, in Spain see: Lozano 1995, pp. 103–129; Nando Rosales and Sanz Ponce 2019, pp. 517–534.

5 United Nations Educational, Scientific and Cultural Organization 1997.

6 See also: Explanations relating to the EU Charter 2007.

7 See: Karran 2007, pp. 289–313; Beiter, Karran and Appiagyei-Atua 2016, pp. 295–306.

8 Karran 2009, p. 191; Rittberger and Richardson 2019, p. 324. See also: Berggren and Bjørnskov 2022a, pp. 1313–1342.

9 Stachowiak-Kudła and Kudła 2017, pp. 1718–1735.

10 See: Bok 2003; Aberbach and Christensen 2018, p. 503 proves that governments ‘are increasingly influencing universities to enact university policies they (the governments) prefer, as are other stake-holders that provide resources’.

11 Barnes 2019, pp. 591–609.

12 Tierney 2001, pp. 7–14; Kayrooz and Preston 2002, pp. 341–358.

13 Safavi and Håkanson 2018, pp. 500–523.

14 Stachowiak-Kudła 2022, pp. 105–106.

15 See: Case C-66/18 European Commission v Hungary (6 October 2020), ECLI:EU:C:2020:792.

16 Palfreyman 2007, pp. 31–35, see also: Ceci, Williams and Mueller-Johnson 2006, pp. 553–569; Suk Gersen 2022, p. 781.

17 Examples of such conflicts have been indicated by: Kors and Silverglate 1998; Ackermann 2020; Cross and Richardson-Self 2020, pp. 31–54; Hayes 2021, pp. 7–14.

18 Berggren and Bjørnskov 2022b, pp. 205–228.

19 For an analysis see: Pogany 1993, pp. 332–355.

20 McKenna 1985, pp. 460–470.

21 Amaral and Carvalho 2003, pp. 35–46.

22 The Fundamental Law of Hungary (April 25, 2011) has replaced the previous constitutional document (The Constitution of the Republic of Hungary, Act XX of 1949).

23 See: Kovács and Tóth 2011, pp. 183–203; Tóth 2012; Bánkuti, Halmai and Scheppele 2019, pp. 1439–1488.

24 The decisions were collected from online legal information systems. The researched period is not equal for each country: Belgium since 1985, Czechia since 1993, France since 1958, Germany since 1973, Hungary since 1990, Italy since 1956, Poland since 1997, Portugal since 1983 and Spain since 1980.

25 According to the accepted scientific views, the first wave took place after World War II in Germany and Italy, the second wave after the collapse of the Spanish and Portuguese authoritarian governments, and of the Greek dictatorship; and the third wave followed the collapse of the Soviet Union in CEE countries. See Ferejohn 2002, pp. 50–51; Stone Sweet 2012, p. 818.

26 For a deeper analysis of the question how these norms are implemented in Hungary and Poland, with particular emphasis on the principle of rule of law, see Ramanujam and Wijenayake 2022, pp. 27–48.

27 For more on these threats see Ignatieff and Roch 2018; Ramanujam and Wijenayake 2022; Ryder 2022

28 Vrielinka, Lemmensa, Parmentiera et al. 2011, pp. 117–141.

29 Berlin 2004a [1969], p. 32, See also: Alexy 2002, p. 141.

30 Stachowiak-Kudła 2020, pp. 25–26; Quinn and Levine 2014, pp. 903–905. See also: Simpson 2020, pp. 287–319; United Nations General Assembly, Report of the Special Rapporteur on the promotion and protection of the right to freedom of opinion and expression, David Kaye, 28 July 2020, A/75/26 para 5.

31 Grimm 2015, p. 16.

32 Basic Law for the Federal Republic of Germany, 23 May 1949.

33 Paragraph 4 (2, 3 and 4) Hochschulrahmengesetz in der Fassung der Bekanntmachung vom 19. Januar 1999.

34 Decision of the Federal Constitutional Court of Germany, Beschluss vom 29. Mai 1973—1 BvR 424/71, 1 BvR 325/72.

35 Decision of the Federal Constitutional Court of Germany, Beschluss vom 29. Mai 1973—1 BvR 1289/78.

36 Decision of the Federal Constitutional Court of Germany, Beschluss vom 29. Mai 1973—1 BvR 424/71, 1 BvR 325/72.

37 Decision of the Federal Constitutional Court of Germany, Beschluss vom 07. Oktober 1980—1 BvR 1289/78.

38 Decision of the Federal Constitutional Court of Germany, Beschluss vom 20. Juli 2010—1 BvR 748/06.

39 Preamble of Act CXXXIX of 2005 on Higher Education.

40 Instead,’the need for a spiritual and intellectual renewal of the nation’ is emphasised.

41 See: Act XXV of 2017 to amend Act CCIV of 2011 on National Higher Education.

42 Preamble of Act XL of 1994 on the Hungarian Academy of Sciences.

43 It should be pointed out that the normative force of pre-2012 landmark decisions of the Hungarian Constitutional Court has not been clarified in post-2012 practice. The Fourth Amendment to the Constitution in 2013 repealed Constitutional Court decisions made before 2012. But the Constitutional Court decided in Decision 13/2013 (VI. 17.) that the court may continue to refer to these decisions in its reasoning if the constitutional rules have not changed or are similar.

44 Decision of the Constitutional Court of Hungary, 34/1994. (VI. 24.), 177, 182.

45 Decision of the Constitutional Court of Hungary, 34/1994. (VI. 24.), 177, 182. The case concerned access to archives of documents which belonged to the Communist Party and were declared by law to be the property of the Hungarian State. Under the relevant legislation dating from 1969 those documents could be consulted only with the permission of the Minister of Culture and Education. The Constitutional Court ruled several provisions of that legislation to be unconstitutional and made it clear that access to the Communist Party documents for the purpose of scientific research must be made possible and guaranteed by the state, within the limits of constitutional provisions.

46 Art 3, Act of 3 July 2018. The Law on Higher Education and Science.

47 Decision of the Constitutional Court of Poland, 25 listopada 2008 r., sygn. akt K 5/08.

48 Decision of the Constitutional Court of Poland, 12 kwietnia 2012 r., sygn. akt. K 30/10.

49 Art 42(2), Constitution of the Portuguese Republic, April 2, 1976. In fact, academic freedom is a cluster-right, which embodies several dimensions: statutory, scientific, pedagogic, administrative, and financial. See Gomes Canotilho and Moreira 2007, pp. 913–914. Still, as the Portuguese Constitutional Court stated, the Constitution. ‘does not bring greater precision to the concept of university autonomy, limits itself to indicating the material domains to which that autonomy applies (scientific, pedagogical, administrative and financial autonomy and autonomy as regards articles of association), and leaves it to the ordinary law to define the specific scope of the concept.’. (Decision of the Constitutional Court of Portugal 491/2008, 8.3). In this case, the question raised was the constitutionality of the interpretation of two rules contained in the legislation on the Status of Civil Service Managerial Staff, whereby members of teaching staff of public universities could be promoted without sitting a competitive examination aimed at assessing their absolute and relative merit. The Court held that ‘University autonomy is a fundamental constitutional guarantee, the subjective scope of which goes beyond the purely institutional level since it also extends to the status of university staff, notably with regard to freedom of research, teaching, thought and pedagogy, in accordance with the Constitution, constituting what is usually designated "academic freedom". However, university autonomy and scientific freedom require that evaluation of teaching proficiency, for career advancement purposes, should continue to be performed by the traditional method, that is to say according to the rule of appraisal of absolute and relative merit.’.

50 Art. 3(2b), Ley 3/2022, de 24 de febrero, de convivencia universitaria.

51 Decision of the Constitutional Court of Spain, STC 5/1981.

52 Decisions of the Constitutional Court of Spain, SSTC 26/1987, 55/1989 and 106/1990.

53 Guthrie, Rachlinski and Wistrich 2007, pp. 1–44.

54 Ali Nasir 2018, p. 299.

55 Grimm 2015, p. 16.

56 Alexy 2015, p. 16.

57 Kumm 2007, pp. 131–166.

58 Decision of the Federal Constitutional Court of Germany, Beschluss vom: 1 März 1978—1 BvR 333/75; 26. Oktober 2004—1 BvR 911/00; 28. Oktober 2008—1 BvR 462/06; 03. September 2014—1 BvR 1195/14.

59 Decision of the Constitutional Court of Poland, 25 listopada 2008 r., sygn. akt K 5/08, V.4.1. The court refers to the position presented by Garlicki 2001

60 Decisions of the Constitutional Court of Spain: STC 5/1981, FJ 7 and AUTO 457/1989, FJ 3.

61 Decision of the Federal Constitutional Court of Germany, Beschluss vom: 17. Februar 2016—1 BvL 8/10.

62 Paragraph 36 Hochschulrahmengesetz in der Fassung der Bekanntmachung vom 19. Januar 1999.

63 Decision of the Federal Constitutional Court of Germany, Beschluss vom 28. Oktober 2008—1 BvR 462/06.

64 Decision of the Constitutional Court of Poland, 25 listopada 2008 r., sygn. akt K 5/08, V.4.1.

65 Decisions of the Constitutional Court of Spain: STC 5/1981, FJ 7 and AUTO 457/1989, FJ 3.

66 Byrne 1989, p. 264.

67 Fuchs 1963, pp. 431–432; DeGeorge, Block, Fuchs, McGee, Rorty and Searle 1997, pp. 54–55.

68 See e.g.: DeGeorge 2003, pp. 11–25; Andreescu 2009, pp. 559–578; Hansson 2019, p. 342.

69 Donnelly 2007, p. 282. See also: Perry 2000, p. 5; Beitz 2009, p. 83; Landman 2016, pp. 1–20; Freeman 2022.

70 Basic Law for the Federal Republic of Germany, 23 May 1949.

71 Paragraph 36 Hochschulrahmengesetz in der Fassung der Bekanntmachung vom 19. Januar 1999.

72 Decision of the Constitutional Court of Poland, 28 kwietnia 2009 r., sygn. akt K 27/07, III.4.4.

73 Ibid. III.4.2.

74 Decision of the Constitutional Court of Hungary, 34/1994. (VI. 24.), 177, 182.

75 Decision of the Constitutional Court of Hungary, 39/2006. (IX. 27.), 498, 501–502.

76 Decision of the Constitutional Court of Portugal, 169/1990, 14.

77 Decision of the Constitutional Court of Spain, STC 179/1996.

78 Decision of the Constitutional Court of Spain, STC 5/1981, FJ 9.

79 Hasnas 2019

80 Stachowiak-Kudła 2021, p. 1036.

81 Decision of the Federal Constitutional Court of Germany, Beschluss vom 28. Oktober 2008 – 1 BvR 462/06.

82 Alexy's theory 2000 and 2002.

83 Klatt and Meister 2012, pp. 687-708; Rivers 2014, p. 413; Jackson 2015, pp. 3094-3196; Ramshaw 2019, pp. 120-142.

84 Decision of the Federal Constitutional Court of Germany, Beschluss vom 28. Oktober 2008 – 1 BvR 462/06.

85 Decision of the Constitutional Court of Spain, STC 179/1996, 12 de noviembre.

86 Stachowiak-Kudła 2021, p. 1036.

87 Berlin (2004b) [1969], pp. 166–217. See also: Kreimer 1984, p. 1315. Berlin’s distinction has been exposed to such a critique. See Simhony 1993, p. 29.

88 Möller 2012, pp. 29–30.

89 Raz 1986, p. 369.

90 Taylor 1979

91 MacCallum, 1967, p. 312.

92 Decision of the Federal Constitutional Court of Germany, Beschluss vom 15 Januar 1958—1 BvR 400/51.

93 Alexy 2015, p. 5.

94 Grimm 2015, p. 18.

95 Alexy 2002, p. 160, 295.

96 Decision of the Federal Constitutional Court of Germany, Beschluss vom: 29. Mai 1973—1 BvR 424/71, 1 BvR 325/72; 1 März 1978—1 BvR 333/75; 26. Oktober 2004—1 BvR 911/00; 28. Oktober 2008—1 BvR 462/06; 20. Juli 2010—1 BvR 748/06; 24. Juni 2014—1 BvR 3217/07; 3. September 2014—1 BvR 1195/14; 12. Mai 2015—1 BvR 1501/13; 26. Juni 2015—1 BvR 2218/13; 17. Februar 2016—1 BvL 8/10.

97 Decision of the Federal Constitutional Court of Germany, Beschluss vom 15. Januar 1958—1 BvR 400/51.

98 Decision of the Federal Constitutional Court of Germany, Beschluss vom 29. Mai 1973—1 BvR 424/71, 1 BvR 325/72.

99 Decision of the Federal Constitutional Court of Germany, Beschluss vom 07. Oktober 1980 – BVerfGE 55, 37–71. See also: Starck 2000, p. 16.

100 Art 70/G(2), Hungarian Constitution; Art X(2), The Fundamental Law of Hungary.

101 The amendment incorporated an additional sentence into the original version of the provision on academic freedom saying that ‘within the framework of the relevant legislation, the Government shall determine the financial structure of the state’s higher educational institutions and the Government shall supervise their financial management’, Art X(3) of the Fundamental Law of Hungary.

102 Concerns in this regard were also expressed in the opinion of the Venice Commission. Opinion on the Fourth Amendment to the Fundamental Law of Hungary Adopted by the Venice Commission at its 95th Plenary Session (Venice, 14–15 June 2013), p. 14.

103 See in particular Act XXXVI of 2014 introducing the ‘chancellor system’, changing the selection of rectors and other relevant modifications (for a more detailed analysis see Kováts 2015, pp. 26–39). Legislative amendments of that kind introduced in the last decade in Hungary were often ‘wrapped’ in efficiency arguments. See f. e. the explanatory notes attached to Act XXXVI of 2014: ‘It is important that the freedom of research and education should allow institutions to operate in a self-governing manner through elected bodies and an elected rector, but *learning from the negative experience of the last 20–25 years of an extensive interpretation of university autonomy* is at least as important, to ensure the responsible, transparent and professional management of institutions … through … professional managers, i.e. chancellors appointed by the government.’.

104 Decision of the Constitutional Court of Hungary, 41/2005. (X. 27.), 459, 475–476.

105 According to the contested provisions, the Minister could dissolve an institution either if it did not comply with the requirement of economic and rational management (for instance if it exceeded its budget) or the entrance examinations were unsuccessful in three consecutive years.

106 Decision of the Constitutional Court of Hungary, 41/2005. (X. 27.), 459, 480. In this context, Ramanujam and Wijenayake describe, as a negative example, a law which came into force in 2019 in Hungary depriving the Hungarian Academy of Sciences of financial and administrative control over its research institutes and giving this power to a newly established governing body, over which the Government has considerable influence (Ramanujam and Wijenayake 2022, 34 –35). The act was challenged by the president of the Academy before the Constitutional Court, and the case is still pending (case no. IV/01399/2019).

107 Decision of the Constitutional Court of Poland, 7 lutego 2006 r., sygn. akt SK 45/04, III.8. See also: 12 kwietnia 2012 r., sygn. akt. K 30/10, III.2.1.

108 Constitution of the Portuguese Republic, April 2, 1976, Art 43(2).

109 Decision of the Constitutional Court of Portugal, 491/2008, 8.3.

110 Decision of the Constitutional Court of Spain, STC 55/1989, FJ 2.

111 Decision of the Constitutional Court of Spain, STC 5/1981.

112 Brand 2018, p. 353.

113 Hill 2011, pp. 3–11.

114 Ben-David 1971, pp. 275–296.

115 Alexy 2000, pp. 294–304.

116 Kumm 2004, p. 585; Alexy 2015, p. 3.

117 Decision of the Federal Constitutional Court of Germany, Beschluss vom: 29. Mai 1973—1 BvR 424/71, 1 BvR 325/72.

118 Decision of the Federal Constitutional Court of Germany, Beschluss vom: 29. Mai 1973—1 BvR 424/71, 1 BvR 325/72; 26. Oktober 2004—1 BvR 911/00; 20. Juli 2010—1 BvR 748/06.

119 Case C-66/18 European Commission v Hungary (6 October 2020), ECLI:EU:C:2020:792, para 227.

120 For a detailed analysis see Bárd 2018, pp. 87–96; Hoxhaj 2021.

121 Case C-66/18, para 228.

122 Case C-66/18, para 228.

123 See: Stachowiak-Kudła 2021, pp. 1031–1048.

124 Decision of Constitutional Court of Hungary, 41/2005.

125 These rules have been introduced by a new law in 2020 which changed the financial and organisational control over the University of Theatre and Film Arts (Budapest). While prior to the legislation the university had been a public institution with decision-making authority vested in an independent senate, through this legislation, ownership over the university has been passed to a private foundation whose Board of Trustees have been appointed by the minister responsible for innovation and technology (and selected from persons closely tied to the government). A transformation of similar nature (so called ‘model change in higher education’) concerned several public universities in Hungary. For more on this issue in a comparative context, see Ramanujam and Wijenayake 2022.

126 Decision of the Constitutional Court of Hungary, 21/2021. (VI. 22.) 6–7, 22–27.

127 Ibid, 44, 46.

128 Ibid, operative part of the decision.

129 Decision of the Constitutional Court of Hungary, 28/2021. (XI. 5.).

130 Practically it means that the semester concerned by the emergency situation does not count as being completed by the students as part of their curriculum. The individual case in which the Constitutional Court’s proceeding was initiated concerns a decision of the funding body of the University of Theatre and Film Arts cancelling the fall semester of 2020. The decision was a ‘response’ to the action of ca. 250 students occupying the campus for more than two months, in protest of the new leadership. For a more detailed analysis see Ryder 2022, 139–159; Kováts 2022.

131 Decisions of Constitutional Court of Poland, 8 listopada 2000 r., sygn. SK 18/99, III.1 and 7 lutego 2006 r., sygn. akt. SK 45/04, III.8.

132 Decision of the Constitutional Court of Portugal, 491/2008, 8.5.

133 Ibid, 8.3. See also: Decisions of the Constitutional Court of Portugal, 169/1990, 14, and 453/2007, II.

134 Decision of the Constitutional Court of Spain, AUTO 42/1992, FJ 2. See also: Botelho 2017, p. 82.

135 Decisions of the Constitutional Court of Spain, STC 55/1989, FJ 2. See also: STC 26/1987, FJ 4; STC 55/1989, FJ 2; STC 106/1990, FJ 6; STC 47/2005, FJ 5; STC 183/2011, FJ 6; STC 176/2015, FJ 4; STC 26/2016, FJ 8; STC 44/2016, FJ 4.

136 Sklansky 2002, p. 1230.

137 MacDonnel 2013, p. 637.

138 Berggren and Bjørnskov (2022b), pp. 205–228.

139 Ibid, p. 220.

140 For a detailed analysis of such a tendency in the post-2012 jurisprudence of the Hungarian Constitutional Court (not only in the area of academic freedom) see Bencze 2022.
